# Association between polygenic risk scores for attention-deficit hyperactivity disorder and educational and cognitive outcomes in the general population

**DOI:** 10.1093/ije/dyw216

**Published:** 2016-09-29

**Authors:** Evie Stergiakouli, Joanna Martin, Marian L Hamshere, Jon Heron, Beate St Pourcain, Nicholas J Timpson, Anita Thapar, George Davey Smith

**Affiliations:** 1 Medical Research Centre (MRC) Integrative Epidemiology Unit (IEU) at the University of Bristol, Bristol, UK,; 2 School of Oral and Dental Sciences, University of Bristol, Bristol, UK,; 3 MRC Centre for Neuropsychiatric Genetics and Genomics, Cardiff University School of Medicine, Cardiff, UK,; 4 School of Social and Community Medicine, University of Bristol, Bristol, UK and; 5 Max Planck Institute for Psycholinguistics, Nijmegen, The Netherlands

**Keywords:** Attention-deficit/hyperactivity disorder (ADHD), polygenic risk scores, Avon Longitudinal Study of Parents and Children (ALSPAC), education, cognitive traits

## Abstract

**Background:** Children with a diagnosis of attention-deficit hyperactivity disorder **(**ADHD) have lower cognitive ability and are at risk of adverse educational outcomes; ADHD genetic risks have been found to predict childhood cognitive ability and other neurodevelopmental traits in the general population; thus genetic risks might plausibly also contribute to cognitive ability later in development and to educational underachievement.

**Methods:** We generated ADHD polygenic risk scores in the Avon Longitudinal Study of Parents and Children participants (maximum *N*: 6928 children and 7280 mothers) based on the results of a discovery clinical sample, a genome-wide association study of 727 cases with ADHD diagnosis and 5081 controls. We tested if ADHD polygenic risk scores were associated with educational outcomes and IQ in adolescents and their mothers.

**Results:** High ADHD polygenic scores in adolescents were associated with worse educational outcomes at Key Stage 3 [national tests conducted at age 13–14 years; β = −1.4 (−2.0 to −0.8), *P* = 2.3 × 10^−6^), at General Certificate of Secondary Education exams at age 15–16 years (β = −4.0 (−6.1 to −1.9), *P* = 1.8 × 10^−4^], reduced odds of sitting Key Stage 5 examinations at age 16–18 years [odds ratio (OR) = 0.90 (0.88 to 0.97), *P* = 0.001] and lower IQ scores at age 15.5 [β = −0.8 (−1.2 to −0.4), *P* = 2.4 × 10^−4^]. Moreover, maternal ADHD polygenic scores were associated with lower maternal educational achievement [β = −0.09 (−0.10 to −0.06), *P* = 0.005] and lower maternal IQ [β = −0.6 (−1.2 to −0.1), *P* = 0.03].

**Conclusions:** ADHD diagnosis risk alleles impact on functional outcomes in two generations (mother and child) and likely have intergenerational environmental effects.

## Introduction

Attention-deficit hyperactivity disorder (ADHD) is the most common childhood neurodevelopmental disorder and is linked to lower cognitive ability^1^ and the need for educational interventions.^2^ In the general population, high levels of ADHD symptoms during early childhood carry risk of worse academic performance^3^ and they are associated with cognitive difficulties years later.^2^ In the longer term, ADHD and the associated difference in academic performance have important negative consequences for employment and earnings in adulthood.^4^

ADHD is a highly heritable complex disorder, with twin study estimates of heritability at 76%.^5^ Genome-wide association studies (GWAS) investigate the contribution of common genetic variants to complex disorders and require large sample sizes to detect the usually small effects of common genetic variants.^6^^,^^7^ GWAS of ADHD have not identified common genetic variants associated at genome-wide level of significance yet, although studies with larger sample sizes are soon to be published (presentation at the World Congress of Psychiatric Genetics, Toronto, 2015).^8^^,^^9^ However, they have indicated that large rare copy number variants (CNVs) can increase risk for ADHD.^10^ Genetic risk scores, known as polygenic risk scores, are a composite score derived from alleles that are associated with a disorder at sub-genome-wide significance thresholds in an independent discovery sample.^11^^,^^12^ They have been used extensively to demonstrate that psychiatric disorders have a polygenic model of inheritance^12^ and shared genetic effects between them.^13^ These genetic risk scores are higher in cases with diagnosed ADHD than in controls^14^ and also contribute to the risk of having higher levels of ADHD traits^15^ and lower cognitive abilities during childhood in the general population.^16^ ADHD as a disorder lies on the spectrum of normal trait variation, and the same risk alleles contributing to ADHD trait levels in the general population also confer risk of ADHD diagnosis, as shown previously using polygenic risk scores.^17^

The current study aimed to examine if ADHD polygenic risk scores predicted adverse educational and cognitive outcomes in adolescents and adults (in mothers of study children) from the general population and to explore if this association was mediated through IQ scores or through ADHD symptoms in childhood.

## Methods

### Discovery sample

The alleles and associated effect sizes selected to be taken forward in order to calculate polygenic risk scores were derived from a case-control UK/Irish ADHD GWAS. This ‘discovery’ study was performed on 727 children aged 4–18 years with a confirmed research and clinical diagnosis of ADHD, and 5 081 population controls from the Wellcome Trust Case Control Consortium–Phase2^18^ analysed on 502 702 single nucleotide polymorphisms (SNPs). Quality control procedures, ascertainment of these samples and GWAS results have been described in detail previously.^9^

### Target sample

The Avon Longitudinal Study of Parents and Children (ALSPAC) is a prospective birth cohort which recruited pregnant women with expected delivery dates between April 1991 and December 1992, from Bristol UK; 14 541 pregnant women were initially enrolled, with 14 062 children born. Detailed information on health and development of children and their parents was collected from regular clinic visits and completion of questionnaires. A detailed description of the cohort has been published previously.^19^^,^^20^ The study website contains details of all the data that are available through a fully searchable data dictionary: [http://www.bris.ac.uk/alspac/researchers/data-access/data-dictionary/]. Ethical approval was obtained from the ALSPAC Law and Ethics Committee and the local ethics committees.

A total of 9912 ALSPAC children and 10 015 ALSPAC mothers were genotyped on the Illumina HumanHap550-quad and the Illumina Human660W-quad genome-wide SNP genotyping platforms, respectively. Quality control procedure details have been published previously.^21^ The resulting data set consisted of 500 527 SNPs for 8365 children and 526 688 for 8340 mothers of European descent, available for analysis.

The discovery sample was selected for this study due to its similarity with the target sample in terms of both ethnicity and geographical location, as well as its robust diagnostic assessment process. Individuals in the target and discovery samples were recruited from geographically nearby regions (South-west England and Wales). An identity by descent (IBD) analysis was conducted using PLINK^22^ to ensure that there were no related individuals between the two samples. Two individuals in the ALSPAC sample who showed an IBD ≥ 12.5% in relation to individuals in the discovery sample were removed from all analyses.

### Phenotypic variables

ADHD traits were assessed in ALSPAC when the participants were aged 7 years and 7 months, using the parent-completed Development and Well-Being Assessment (DAWBA).^23^ Total IQ scores were collected when ALSPAC probands were 15.5 years old, using the computerized version of the Wechsler Abbreviated Scale of Intelligence (WASI)^24^ during a regular clinic visit.

Academic performance for the children was assessed at three different points during their education in the UK. First, Key Stage 3 national tests take place in the UK when children are aged 13–14 years and include tests in English, Maths and Science. A continuous score (0–141) reflecting total points summed over all the national tests at Key Stage 3 was used. Second, the General Certificate of Secondary Education (GCSE) examinations are taken when pupils turn 16 years of age in the UK. Pupils study up to 12 subjects (8 on average), some of which (e.g. English and Maths) are compulsory as part of the National Curriculum. The subjects are graded individually on a scale of A^*^ (highest) to G (lowest). For this analysis, a continuous score (0–483) reflecting capped total points summed over the eight best GCSE grades achieved was employed. ^25^ Third, Key Stage 5 examinations are taken when the children are 16–18 years of age (A levels) to apply to university. A categorical variable was used to assess whether children with available GCSE examination results continued to take Key Stage 5 tests.

Information on maternal education was collected by questionnaires completed when the mothers were in the 32^nd^ week of gestation. The mothers were asked about all the qualifications they had obtained. From the data, a 5-point education scale was obtained, with the following categories: No qualifications/No higher than GCSE (D, E, F or G); Vocational qualification; O-Level or equivalent; A-level or equivalent; Teaching or Nursing qualification/University degree.

A subset of ALSPAC mothers completed the Wechsler Abbreviated Scale of Intelligence (WASI)^24^ while accompanying their children to a clinic visit when the children were 15.5 years old, from which total IQ scores were calculated.

### Statistical analysis

#### Polygenic risk score analysis

Polygenic risk scores were calculated separately for ALSPAC children and mothers according to the method described by the International Schizophrenia Consortium (ISC)^12^ based on the results of the independent discovery sample. In line with previous studies,^11^^,^^12^^,^^26^ a threshold of *P* < 0.5 was used to select alleles more common in cases than controls from the discovery sample (SNPs in relative linkage equilibrium in the ALSPAC GWAS were first selected). These identified SNPs were used to calculate a polygenic score for each individual in ALSPAC, corresponding to the mean number of score alleles (weighted by the logarithm of the odds ratio) across the set of SNPs. Analysis was performed using PLINK.^22^

Multivariate linear regression models were used to test whether standardized polygenic risk scores for ADHD were associated with total IQ and educational outcomes including gender as a covariate. The same analysis was also performed when stratifying children by sex, due to the higher numbers of boys with ADHD. When there was an apparent difference in effect magnitude between boys and girls, a test of interaction was performed by including an interaction term in the model. Logistic regression was employed for categorical variables. The amount of variance explained was calculated as the difference of Nagelkerke pseudo-R^2^ in the full model, as compared with the null model which included sex but not polygenic score. *P*- values were determined from likelihood ratio tests, which compare the full model with the null model.

#### Structural equation modelling

Educational achievement and cognitive ability as measured by IQ scores are strongly correlated, and structural equation modelling (SEM) can incorporate the association of ADHD polygenic risk scores with both outcomes, to unpick pleiotropic and unique relationships with each outcome. The Sobel–Goodman mediation test^27^^,^^28^ was used to calculate direct and indirect effects of ADHD polygenic risk scores on education and IQ/ADHD symptoms, with educational achievement as the outcome, and IQ or ADHD symptoms as the mediator in ALSPAC children. Sex was included in this model as a covariate. However, in the case where the product of coefficients *α* and *β* in Sobel–Goodman test is not normally distributed, the standard errors of the indirect effect will not be accurately calculated.^29^^,^^30^ For this reason, we also performed 1000 bootstrap replications (that do not rely on the assumption of normality)^31^ by resampling the dataset to create an empirical approximation of the sampling distribution of the indirect effect. We present the results from both types of analyses.

Multiple mediation, including both IQ and ADHD symptoms as mediators in the model, was performed using Zellner’s seemingly unrelated regression,^32^ and 1000 bootstrap replications by resampling the dataset to obtain accurate standard errors for the beta coefficients. Mediation testing was performed in Stata v13.1.^33^

## Results

A total of 5748 children from ALSPAC (51.1% male, *n* = 2942 and 48.9% female, *n* = 2806) had genetic data and complete DAWBA data assessed at an average age of 7 years and 7 months [standard deviation (SD) 1.7 months]; 114 children had a DSM-IV diagnosis of ADHD (any type) based on the DAWBA. A total of 4958 children from ALSPAC (47.6% male, *n* = 2363 and 52.4% female, *n* = 2595) had genetic data and full-scale IQ data from the WASI assessment at an average age of 15 years and 5 months (SD 3 months). Their mean full-scale IQ was 91.9 (SD 13). In all, 6385 children had both genetic data and Key Stage 3 data, with mean Key Stage 3 score of 106.2 points (SD 24.4), and 6928 children had both genetic data and GCSE data with mean score of 328.2 points at GCSE level (SD 89.9). Of those who sat GCSEs, 2782 did not continue to Key Stage 5 level and 4146 did.

A total of 3319 mothers from ALSPAC had genetic and IQ WASI data. Their mean age was 45 years and 4 months at completion of the IQ test (SD 4 years and 5 months) and their mean full-scale IQ was 98.9 (SD 13.9).

Polygenic risk scores for ADHD were associated with worse adolescent educational outcomes, as indicated by the score achieved at the end of Key Stage 3 as well as by GCSE scores (Table 1). ADHD polygenic risk scores were also associated with lower odds of sitting Key Stage 5 examinations among those who sat GCSE exams [OR = 0.90 per unit increase in polygenic score (0.88 to 0.97), *P* = 0.001]. Moreover, the results show an association of ADHD polygenic risk scores with decreased IQ at age 15.5 years [β = −0.8 units of IQ per unit increase in polygenic score (−1.2 to −0.4), *P* = 2.4 × 10^−4^] (Table 1). Sex-stratified analysis did not point to different effects between boys and girls (Supplementary Table 1, available as Supplementary data at *IJE* online).

**Table 1.** Associations of childhood ADHD polygenic risk scores with educational outcomes and IQ at age 15.5 years

Outcome	N	Beta coefficient (95% CIs)	*P*-value	R^2^
IQ age 15.5 years	3858	−0.8 (−1.2 to −0.4)	2.4 × 10^−4^	0.003
Key Stage 3 points	6385	−1.4 (−2.0 to −0.8)	2.3 × 10^−6^	0.003
Capped GCSE points	6928	−4.0 (−6.1 to −1.9)	1.8 × 10^−4^	0.006

CIs, Confidence Intervals; GCSE, General Certificate of Secondary Education

In ALSPAC, maternal high genetic loading as indicated by ADHD polygenic risk scores was associated with lower maternal educational achievement [β = −0.09 points in the education scale (−0.10 to −0.06), *P* = 0.005], as measured by the mothers’ self-reported highest qualification obtained and lower maternal IQ [β = −0.6 (−1.2 to −0.1), *P* = 0.03; and Table 2].

**Table 2.** Associations of maternal ADHD polygenic risk scores with maternal educational achievement and IQ

Outcome	N	Beta coefficient (95% CIs)	P value	R^2^
Mother’s highest qualification	7280	−0.09 (−0.10 to −0.06)	0.005	0.005
Maternal total IQ	2313	−0.6 (−1.2 to −0.1)	0.030	0.002

CIs, Confidence Intervals

Maternal ADHD polygenic risk scores were also associated with worse educational outcomes in their offspring at both Key Stage 3 and GCSE exams (Table 3). Offspring ADHD polygenic score was included in the model to account for the correlation of ADHD polygenic scores in mothers and their adolescent offspring (Pearson’s correlation coefficient: 0.46). Stronger effects were observed when offspring polygenic score was included in the models, indicating that there is a genetic as well as environmental component in the association. In addition, polygenic scores for ADHD in adolescents were associated with lower maternal educational achievement [β = −0.06 (−0.10 to −0.02), *P* = 0.002] when adjusting for maternal ADHD polygenic scores (Table 3).

**Table 3.** Associations of maternal polygenic risk scores (PGS) with childhood educational outcomes and childhood polygenic risk scores with maternal educational outcomes

Outcome	Predictor	N	Beta coefficient (95% CIs)	*P*-value	R^2^
Key Stage 3 points	Maternal PGS adjusted for childhood PGS	4239	−1.1 (−1.8 to −0.3)	0.007	0.02
Key Stage 3 points	Maternal PGS	4239	−1.4 (−2.1 to −0.9)	1.7 × 10^−6^	0.02
Capped GCSE points	Maternal PGS adjusted for childhood PGS	4263	−2.9 (−5.7 to −0.2)	0.036	0.03
Capped GCSE points	Maternal PGS	4263	−4.3 (−6.4 to −2.1)	9 × 10^−5^	0.03
Mother’s highest qualification	Childhood PGS	5004	−0.06 (−0.10 to −0.02)	0.002	0.008

CIs, Confidence Intervals; PGS, Polygenic Risk Score; GCSE, General Certificate of Secondary Education

### 

#### Exploratory mediation analysis

**Figure 1.**
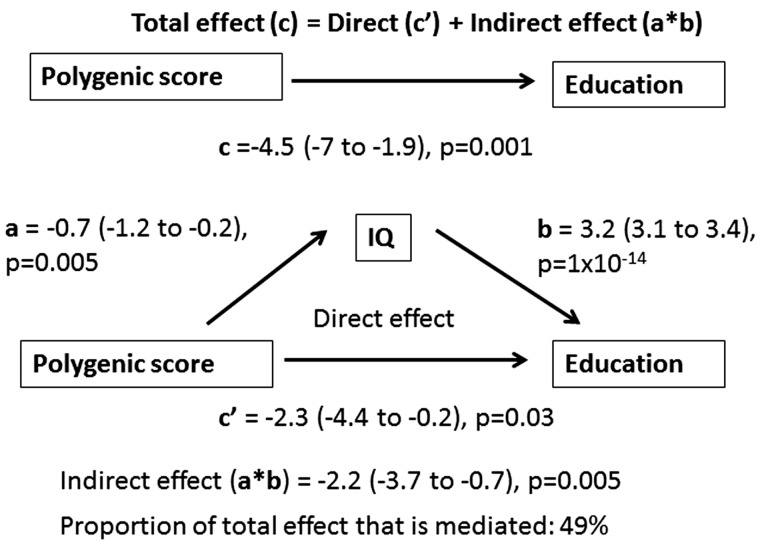
Structural Equation Modelling Analysis Based on Sobel-Goodman Test of Mediation^22,23^ in ALSPAC Children with GCSE Results as Outcome and IQ as Mediator.

**Figure 2.**
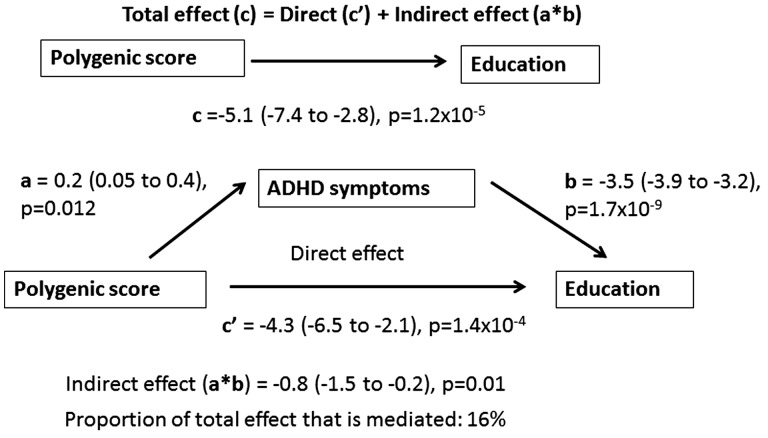
Structural Equation Modelling Analysis Based on Sobel-Goodman Test of Mediation^22,23^ in ALSPAC Children with GCSE Results as Outcome and ADHD symptoms as Mediator.

To explore if the association between children’s ADHD polygenic scores on educational outcomes is mediated through their IQ scores or through their ADHD symptoms in childhood, we performed structural equation modelling using the Sobel-Goodman test. In the first model, including GCSEs results as the outcome and IQ as the mediator, 49% of the effect of ADHD polygenic scores on educational outcomes in adolescents was mediated through IQ (Figure 1). In the second model, ADHD symptoms instead of IQ were used as the mediator. There was evidence of mediation with 16% of the total effect of ADHD polygenic scores on GCSE educational outcomes mediated through ADHD symptoms (Figure 2). For both mediators, the associations were similar when Key Stage 3 results were modelled as the outcome (Supplementary Figures 1 and 2, available as Supplementary data at *IJE* online). Results from the mediation analysis using bootstrapping showed a consistent pattern of results and are presented in Supplementary Tables 2 and 3 (available as Supplementary data at *IJE* online).

A model with both IQ and ADHD symptoms as mediators was also tested. This model supported evidence for an indirect effect [−2.5 (−4.1 to −0.9), *P* = 0.002] with most of the mediated effect (79%) attributed to IQ (Supplementary Figure 3, results from bootstrapping in Supplementary Table 4, available as Supplementary data at *IJE* online).

## Discussion

Our findings demonstrate, for the first time, shared genetic overlaps between childhood ADHD and functional outcomes (educational attainment, IQ) not only in adolescents but also in a different generation: an association between educational attainment and lower IQ scores with ADHD polygenic scores was found for mothers in this sample. Exploratory analysis suggests that the association of ADHD polygenic risk scores with educational outcomes in children is mediated substantially but not entirely by IQ and to a lesser extent by earlier levels of ADHD symptoms.

Previous studies using a different approach found that polygenic risk scores for educational attainment and IQ predicted ADHD traits.^34^^,^^35^ These studies support a genetic overlap of ADHD symptoms in the general population and educational achievement, at least in terms of common genetic risk variants, which needs to be further investigated. Our study extends previous findings for adolescents from the general population, showing that ADHD genetic risk impacts on poorer educational and cognitive outcomes in two generations (mother and child) and likely has intergenerational environmental effects.

Genetic and environmental influences on psychopathology are often correlated in a phenomenon known as gene-environment correlation.^36^^,^^37^ Since parents provide both genetic factors and the rearing environment to their children, it is often the case that parental genetic factors are correlated with environmental factors in the rearing environment.^38^ The associations between maternal ADHD polygenic risk scores and offspring educational attainment observed in this study could represent such an instance of passive gene-environment correlation. Mothers with high ADHD polygenic scores maybe more likely to have a higher number of ADHD and other symptoms themselves, and this could directly influence the educational level that their children can achieve, or increase exposures to early adversities that in turn impact on adolescent educational outcomes.

Based on these results, we can hypothesize about the mechanism through which high genetic loading for ADHD might lead to impaired academic performance. One possibility is that risk variants for ADHD can increase the number of ADHD symptoms and other neurodevelopmental difficulties^15^^,^^16^ in the general population, and this in turn could interfere with educational achievement. However, mediation analysis pointed to an indirect effect of ADHD polygenic scores on education through IQ, which implies pleiotropic effects of common genetic variants on several behavioural and cognitive traits in the general population. We note that this is a preliminary, exploratory analysis. Structural equation modelling requires its assumptions to be met by all variables in the diagram.^39^^,^^40^ Differential measurement error between the mediator and the exposure can reduce power and lead to invalid conclusions in this type of analysis.^41^^,^^42^ More importantly, whatever the underlying mechanism of the reported associations, this study highlights the importance of recognizing that ADHD risks contribute to later functional outcomes. The finding that the association of ADHD polygenic scores with educational under-achievement and lower IQ scores was observed also in mothers suggests that the potential benefits of any educationally-focused interventions in childhood could translate into better outcomes in adult life.

The most important advantage of our study lies with the use of appropriate samples as a discovery and target population. The discovery sample was a well-characterized sample of children with an ADHD diagnosis confirmed by a semi-structured research diagnostic interview.^9^ The availability of summary statistics, including effect sizes, meant that weighted polygenic scores could be calculated in an independent sample. The target population benefited from a large sample of children representative of the population and their mothers, who have been followed for a long period of time. Both samples were collected from geographically near regions and appropriate analysis was performed to exclude any individuals who were potentially related between the two samples. The phenotypes analysed were based on well-established tests, and the educational outcomes were assessed using real-life examination results obtained though linkage of participants’ information with school databases after consent was granted.^19^^,^^20^

The main limitation of this study is that the ADHD polygenic scores explained a limited amount of variance in phenotypes tested in the general population. This is true for complex disease polygenic risk scores. Also, GWAS of ADHD are still small and thus of limited power, when compared with GWAS of other psychiatric disorders.^8^^,^^9^ In addition, since the ALSPAC sample is a population cohort, the majority of participants will not be scoring in the negative extremes of the distribution for IQ and educational outcomes.

In conclusion, we found that higher genetic loading for clinical ADHD is associated with functional outcomes in adolescents from the general population and in their mothers. Further investigation is required to determine the mechanisms that account for these links, so that children with ADHD symptoms and ADHD risk can benefit from appropriate interventions and support to achieve their potential in education.

## Supplementary Data

Supplementary data are available at *IJE* online.

## Funding

This work was supported by the Medical Research Council and the University of Bristol [MRC Integrative Epidemiology Unit (IEU) MC_UU_12013/1‐9]; the Medical Research Council, the Wellcome Trust and Cardiff University [MRC Centre for Neuropsychiatric Genetics and Genomics 079711/Z/06/Z]; the Medical Research Council, the Wellcome Trust and the University of Bristol [core ALSPAC support 102215/2/13/2].

**Key messages**

- The contribution of psychiatric genetic risks to functional outcomes in adulthood has not previously been investigated.
- High ADHD polygenic scores are associated with worse educational outcomes and lower IQ in adolescents from the general population and in their mothers.
- ADHD genetic risk impacts on poorer educational and cognitive outcomes in two generations (mother and child) and likely has intergenerational environmental effects.

## Supplementary Material

Supplementary Data
