# Supplementary material for: Association between polygenic risk scores for attention-deficit hyperactivity disorder and educational and cognitive outcomes in the general population
Source: Int J Epidemiol. 2016 Sep 29;46(2):421–8. doi: 10.1093/ije/dyw216 (PMC5424076; doi:10.1093/ije/dyw216)
Supplement: Supplementary Data [file dyw216_supp.docx]

**Web Appendix**

**Supplementary Table 1.** Associations of Childhood ADHD Polygenic Risk Scores with Educational Outcomes and IQ at Age 15.5 Years – Sex Stratified Analysis

| Outcome | N | Beta Coefficient (95% CIs) | P value | R^2^ |
| --- | --- | --- | --- | --- |
| Total point score achieved at key stage 3 | Boys (3250) | -1.7 (-2.6 to -0.8) | 1.7x10^-4^ | 0.004 |
|  | Girls (3135) | -1.2 (-2.0 to -0.4) | 4.2x10^-3^ | 0.003 |
| Capped GCSE points | Boys (3498) | -4.8 (-8.0 to -1.7) | 2.4x10^-3^ | 0.003 |
|  | Girls (3430) | -3.1 (-5.9 to -0.3) | 2.9x10^-2^ | 0.001 |
| Total IQ at age 15.5 | Boys (1845) | -0.2 (-0.8 to -0.4) | 0.53 | 0.002 |
|  | Girls (2013) | -1.1 (-1.7 to -0.6) | 1x10^-4^ | 0.008 |

GCSE, General Certificate of Secondary Education.

**Supplementary Table 2.** Structural Equation Modelling Analysis of ADHD Polygenic Risk scores with Education as the Outcome and IQ as the Mediator in ALSPAC children using 1000 Bootstrap Replications

| Type of effect | Beta Coefficient^*^ | N |
| --- | --- | --- |
| Indirect effect with GCSEs as the educational outcome | -2.2 (-3.8 to -0.7) | 2864 |
| Direct effect with GCSEs as the educational outcome | -2.30 (-4.30 to -0.04) | 2864 |
| Indirect effect with Key Stage 3 results as the educational outcome | -0.6 (-1.1 to -0.2) | 2563 |
| Direct effect with Key Stage 3 results as the educational | -0.8 (-1.4 to -0.2) | 2563 |

*Note that percentile confidence intervals for beta coefficients are reported.

GCSE, General Certificate of Secondary Education.

**Supplementary Table 3.** Structural Equation Modelling Analysis of ADHD Polygenic Risk Scores with Education as Outcome and ADHD Symptoms as Mediator in ALSPAC Children Using 1000 bootstrap replications

| Type of effect | Beta Coefficient^*^ | N |
| --- | --- | --- |
| Indirect effect with GCSEs as the educational outcome | -0.8 (-1.5 to -0.2) | 4824 |
| Direct effect with GCSEs as the educational outcome | -4.2 (-6.5 to -2.2) | 4824 |
| Indirect effect with Key Stage 3 results as the educational outcome | -0.20 (-0.40 to -0.02) | 4390 |
| Direct effect with Key Stage 3 results as the educational | -1.3 (-2.0 to -0.7) | 4390 |

*Note that percentile confidence intervals for beta coefficients are reported.

GCSE, General Certificate of Secondary Education.

**Supplementary Table 4.** Multiple Mediation Model of ADHD Polygenic Risk Scores on Educational Outcomes (GCSEs) with Both IQ at Age 15.5 Years and ADHD Symptoms as Mediators Using 5000 Bootstrap Replications

| Type of effect | Beta Coefficient | P value | N |
| --- | --- | --- | --- |
| Indirect effect through IQ | -2.0 (-3.3 to -0.6) | 0.005 | 2841 |
| Indirect effect through ADHD symptoms | -0.5 (-1.5 to 0.4) | 0.3 | 2841 |
| Total indirect effect | -2.5 (-4.3 to -0.8) | 0.005 | 2841 |

GCSE, General Certificate of Secondary Education.

**Supplementary Figure 1**. Structural Equation Modelling Analysis Based on Sobel-Goodman Test of Mediation^1,2^ in ALSPAC Children with Key Stage 3 Results as Outcome and IQ as Mediator.


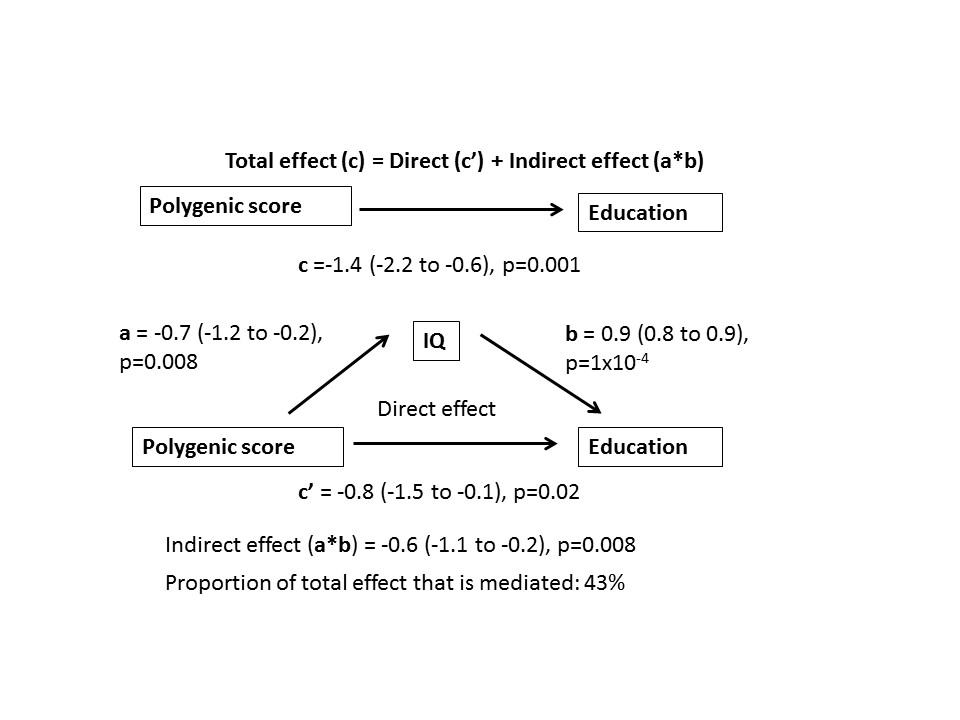


The total effect (c) of ADHD polygenic scores on education (Key Stage 3 results) is the sum of the direct effect (c’) and the indirect effect of ADHD scores on education through IQ at age 15.5 (a*b). The indirect effect (a*b) is the product of the coefficients of the effects of polygenic score on IQ (a) and the effect of IQ on education (b). Beta coefficients with 95% CIs and p values from the Sobel-Goodman test are presented. N=2563. Abbreviation: ALSPAC, Avon Longitudinal Study of Parents and Children.

**Supplementary Figure 2.** Structural Equation Modelling Analysis Based on Sobel-Goodman Test of Mediation^1,2^ in ALSPAC Children with Key Stage 3 Results as Outcome and ADHD Symptoms as Mediator.


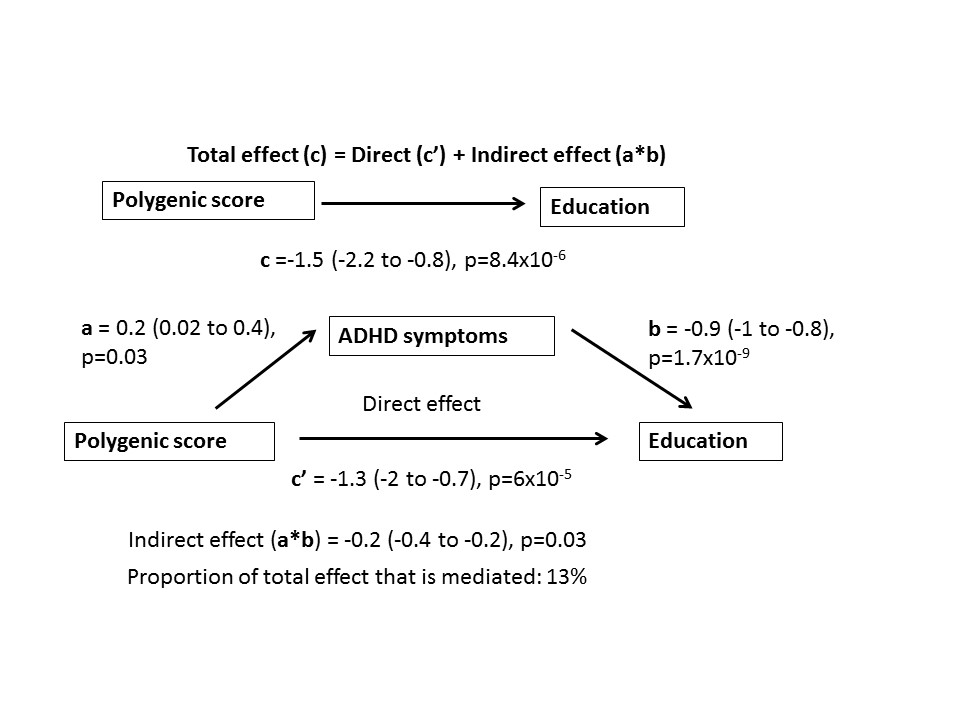


The total effect (c) of ADHD polygenic scores on education (Key Stage 3 results) is the sum of the direct effect (c’) and the indirect effect of ADHD scores on education through the number of ADHD symptoms (a*b). The indirect effect (a*b) is the product of the coefficients of the effects of polygenic score on ADHD symptoms (a) and the effect of ADHD symptoms on education (b). Beta coefficients with 95% CIs and p values from the Sobel-Goodman test are presented. N=4390. Abbreviation: ALSPAC, Avon Longitudinal Study of Parents and Children.

**Supplementary Figure 3.** Multiple Mediation Analysis Using Zellner’s Seemingly Unrelated Regressions^3^ with GCSE Results as Outcome and Both IQ and ADHD Symptoms as Mediators in ALSPAC Children.


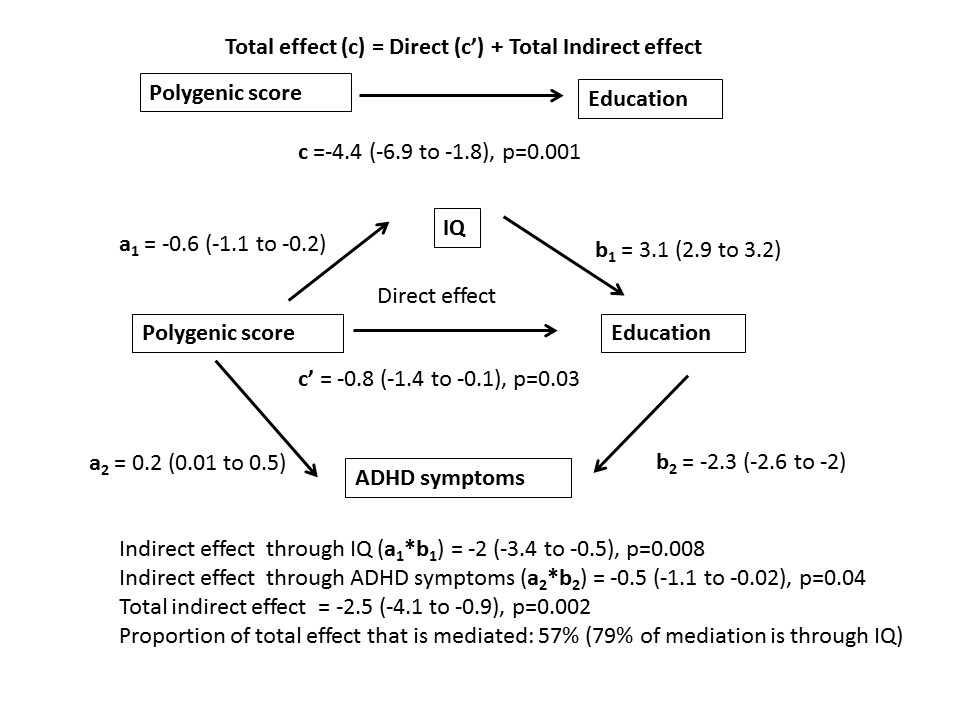


The total effect (c) of ADHD polygenic scores on education (GCSE results) is the sum of the direct effect (c’) and the total indirect effect of ADHD scores on education through IQ and the number of ADHD symptoms (a*b). The total indirect effect is the sum of the indirect effect of IQ and the indirect effect of ADHD symptoms. N=2841. Abbreviation: ALSPAC, Avon Longitudinal Study of Parents and Children.

**References**

1. Sobel ME. Some new results on indirect effects and their standard errors in covariance structure models. *Sociological Methodology.* 1986;16:159-186.

2. Sobel ME. Asymptotic confidence intervals for indirect effects in structural equation models. *Sociological Methodology.* 1982;13:209-312.

3. Preacher KJ, Hayes AF. Asymptotic and resampling strategies for assessing and comparing indirect effects in multiple mediator models. *Behav Res Methods.* 2008;40(3):879-891.
